# Superradiance of bacteriochlorophyll *c* aggregates in chlorosomes of green photosynthetic bacteria

**DOI:** 10.1038/s41598-021-87664-3

**Published:** 2021-04-16

**Authors:** Tomáš Malina, Rob Koehorst, David Bína, Jakub Pšenčík, Herbert van Amerongen

**Affiliations:** 1 Department of Chemical Physics and Optics, Faculty of Mathematics and Physics, Charles University, Prague, Czech Republic; 2 Laboratory of Biophysics, Wageningen University, Wageningen, The Netherlands; 3 MicroSpectroscopy Research Facility, Wageningen University, Wageningen, The Netherlands; 4 Faculty of Science, University of South Bohemia, České Budějovice, Czech Republic; 5 Biology Centre, Czech Academy of Science, České Budějovice, Czech Republic

**Keywords:** Biophysics, Optics and photonics

## Abstract

Chlorosomes are the main light-harvesting complexes of green photosynthetic bacteria that are adapted to a phototrophic life at low-light conditions. They contain a large number of bacteriochlorophyll *c*, *d*, or *e* molecules organized in self-assembling aggregates. Tight packing of the pigments results in strong excitonic interactions between the monomers, which leads to a redshift of the absorption spectra and excitation delocalization. Due to the large amount of disorder present in chlorosomes, the extent of delocalization is limited and further decreases in time after excitation. In this work we address the question whether the excitonic interactions between the bacteriochlorophyll *c* molecules are strong enough to maintain some extent of delocalization even after exciton relaxation. That would manifest itself by collective spontaneous emission, so-called superradiance. We show that despite a very low fluorescence quantum yield and short excited state lifetime, both caused by the aggregation, chlorosomes indeed exhibit superradiance. The emission occurs from states delocalized over at least two molecules. In other words, the dipole strength of the emissive states is larger than for a bacteriochlorophyll *c* monomer. This represents an important functional mechanism increasing the probability of excitation energy transfer that is vital at low-light conditions. Similar behaviour was observed also in one type of artificial aggregates, and this may be beneficial for their potential use in artificial photosynthesis.

## Introduction

Green sulfur bacteria are capable to live phototrophically at extremely low-light conditions^1,2^. Their light-harvesting system absorbs light and transfers the excitation energy towards the reaction centres with efficiency of more than 80%^3^. This is a rather high value taking into account the complexity of the photosynthetic machinery of green sulfur bacteria^4^. In the reaction centres, the excitation energy is used to generate a charge separation completing the first step of the light energy conversion into the chemical form. The primary light-harvesting complex (LHC) of green bacteria is a chlorosome^5–7^, the largest known photosynthetic antenna. A typical chlorosome is an oblong body of ~ 100–200 × 30–70 × 10–40 nm in size, and is attached to the inner side of the cytoplasmic membrane, where the reaction centres are located.

Chlorosomes are found in three bacterial phyla^6,7^ and here we study members of two of them: filamentous anoxygenic bacterium *Chloroflexus* (*Cfl.*) *aurantiacus* and green sulfur bacterium *Chlorobaculum* (*Cba*.) *tepidum*, both containing BChl *c* as a main pigment*.* Chlorosomes of green sulfur bacteria, and to some extent also green filamentous bacteria, are known to exhibit an efficiency of excitation energy transfer that is redox dependent. *Chlorobi* species are obligate anaerobes and the excited states of bacteriochlorophyll (BChl) *c* aggregates are quenched at aerobic conditions, most probably to avoid formation of damaging photo-oxidative products^8^. A key role in the excitation quenching is played by quinone molecules^9^ and the effect can also be induced in artificial aggregates^10^.

While in all other LHCs the positions and orientations of the pigments are dictated by a protein scaffold, BChl *c*, *d*, or *e* molecules in the chlorosome interior self-assemble into large aggregates. As a result, the pigment concentration in a chlorosome (> 1.5 M)^11^ is much larger than in any other photosynthetic LHC^12^, and yet does not lead to any appreciable self-quenching, and instead leads to the formation of charge-transfer states^13^. Also the number of pigments per reaction centre is the largest known. The strong exciton interactions between closely packed pigments lead to a redshift of the Q_y_ and Soret bands as compared to monomeric BChl *c*. This is important for tuning the absorption into the spectral range of available light. The coupling between pigments also leads to exciton delocalization. The present disorder limits the areas with strong coupling to "coherent domains" and the excitation is initially delocalized over (a part of) the domain^14^. The excitation energy is then supposedly transferred by incoherent hopping between domains, which act as supermolecules and effectively decrease the number of energy transfer steps needed to reach the reaction centre, and thus contribute to the high efficiency^15^. The extent of delocalization is the largest at the moment of excitation into the absorption maximum, because the dipole strengths of these transitions are larger than that of the emitting states (see below). The delocalization decreases in time as a result of exciton relaxation to the emitting states with lower dipole strengths, and due to dynamic disorder^16–18^. Various estimates of the delocalization length at the time of excitation were obtained from transient absorption measurements and values of about 10 molecules were determined for *Cfl. aurantiaucus* and 2–3 for *Cba. tepidum*^19–21^. In this work we address the question what the extent of delocalization is after exciton equilibration. Equilibrium delocalization lengths can be estimated from superradiant enhancement of fluorescence radiative rates^18^.

The aggregation of chlorosomal BChls also leads to a decrease in the fluorescence quantum yield and shortening of the excited-state lifetime^22–24^. It is interesting to compare these properties of BChl *c* aggregates with those of J-aggregates^25^. Transition dipole moments of J-aggregates are oriented in a "head-to-tail" way, which leads to a similar redshift of absorption and lifetime shortening. However, in contrast to BChl *c* aggregates, the fluorescence quantum yield increases upon aggregation. This is a consequence of the excitonic coupling between the chromophores forming the J-aggregate, which results in a large transition dipole moment of the emitting states. The radiative rate is proportional to the number of chromophores in a coherent domain^26^. This effect is called collective spontaneous emission or superradiance^26^. In J-aggregates all the dipole strength is concentrated in the transition to the lowest state which leads to relatively strong superradiance. The delocalization length was estimated to be between only a few up to ~ 50 000 monomers for J-aggregates, depending on the sample form, temperature and other parameters (^27^and references therein) and the delocalization leads to a narrow absorption band via exchange narrowing.

The decrease of the quantum yield in BChl *c* aggregates is related to a redistribution of the oscillator strength upon aggregation. The main absorbing states do not correspond to the lowest exciton level as in J-aggregates, as indicated by the extremely large Stokes shift between the maximum of absorption and fluorescence observed for chlorosomes (~ 30 nm, ~ 500 cm^−1^ for BChl *c* containing bacteria). Another difference compared to J-aggregates is the lack of exchange narrowing. In fact, the absorption bands of aggregated pigments are broader than monomeric bands. Spectrally narrow bands of J-aggregates would not be suitable for light harvesting, and their very high fluorescence quantum yield in combination with a short lifetime would not be suitable for efficient excitation energy transfer (EET). On the other hand, the magnitude of the dipole moment determines the probability of EET, and therefore a small dipole strength would not be suitable as well.

Superradiance was previously observed for strongly coupled pigments in LHCs of purple bacteria^28^, but not for weakly coupled pigments in LHC II from higher plants^29^. Here we investigate how large the emitting dipole strength is in the chlorosomes, which is relevant for excitation-energy transfer between coherent domains. Chlorosomes serve as an inspiration source for artificial light-harvesting complexes for new ways of solar energy utilization. This effort is motivated by their extraordinary light-harvesting properties and the fact that the formation of the pigment aggregates inside the chlorosome is based on self-assembly and can be mimicked in vitro^30–32^. Therefore, we also included artificial aggregates in our study. We show that despite the decrease of the fluorescence quantum yields induced by aggregation, the shortening of the lifetime is even larger and as a consequence the BChl *c* aggregates in the chlorosomes turn out to be superradiant. Chlorosomes and artificial aggregates thus uniquely combine two seemingly contradictive properties. The fluorescence quantum yield of the aggregates is lower than for a monomer, so the probability that the excitation will be lost by fluorescence is also low. At the same time the emitting dipole strength is larger than that of the monomer, which increases the probability of excitation energy transfer. The experiments thus reveal key physical elements that lie at the basis of the exceptional light-harvesting capabilities of chlorosomes and also artificial aggregates.

## Results and discussion

**Figure 1:**
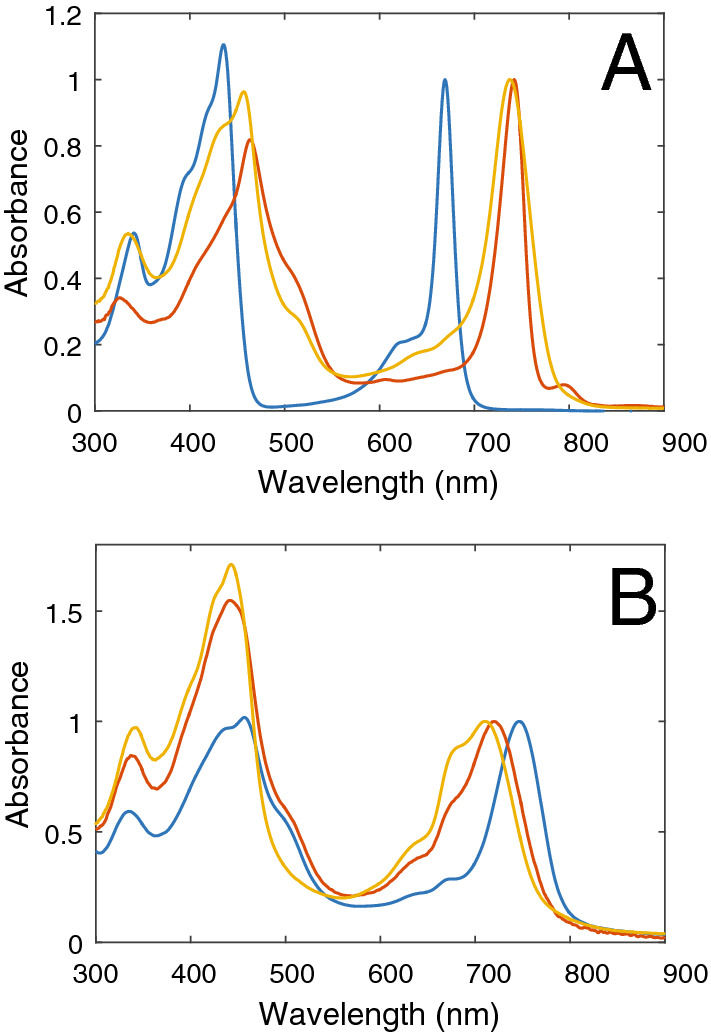
Absorption spectra of (**A**) BChl *c* monomers in ethanol (blue line) and aggregates in chlorosomes from *Cfl.aurantiacus* (red) and *Cba. tepidum* (yellow) in a buffer, and (**B**) pure BChl *c* injected to a buffer by the "fast" method (which forms probably dimers^33^) (yellow) and aggregates prepared by the "fast" (red) and "slow" (blue) method with BChl *c* to β-carotene stoichiometric ratio of 1:0.3. All spectra were normalized to the maximum of the Q_y_ band.

Figure 1 shows absorption spectra of different forms of BChl *c*. Monomeric pigments dissolved in ethanol exhibit a Q_y_ band at ~ 670 nm, which shifts to ~ 740 nm upon aggregation. This redshift is similar in both types of chlorosomes studied in this work. The broader Q_y_ band observed for *Cba. tepidum* reflects a larger structural disorder compared to *Cfl. aurantiacus*. The blue-green region is dominated by the Soret band, which overlaps with a contribution from carotenoids in both types of chlorosomes. The artificial aggregates were prepared in two different ways. First, by rapid injection of pigments dissolved in organic solvent into an aqueous buffer. In this case aggregation of BChl *c* occurs only when a suitable non-polar molecule is added to the initial mixture^33^. Here we used β-carotene, which also transfers excitation energy to BChl *c* and thus extends the absorption spectrum of the artificial aggregate^31^. The redshift is proportional to the carotene content and smaller than in chlorosomes if no other lipophilic component is used^31,33^. The second method is based on slow addition of the buffer into the solution of BChl *c* with a block copolymer^34^. In this case, the polymer induces aggregation and the redshift is similar to that of chlorosomes and independent of the β-carotene content. Although it was not required, we used β-carotene also in this case to allow for a direct comparison of both types of aggregates.

Since the highest molar ratio between carotenoids and BChl *c* is about 0.3 for chlorosomes from *Cfl. aurantiacus*, we compare the results for chlorosomes with artificial aggregates with a similar ratio. Results for other ratios between 0 and 1.0 are shown in Supplementary material. The larger redshift of the aggregates prepared by the "slow" method as compared to that of aggregates prepared with the "fast" method may either be due to stronger excitonic interactions between the BChl *c* molecules, or due to a substantially smaller size of the coherent domains in fast-method aggregates. Thus both natural and artificial BChl *c* aggregates exhibit a redshift caused by strong excitonic interactions between the pigments. As mentioned in the Introduction, the excitonic interactions lead to exciton delocalization. To find out the minimum extent of exciton delocalization of the states responsible for the fluorescence emission, one needs to measure and compare the fluorescence quantum yield ϕ_*fl*_ and lifetime τ_*fl*_ which provide the radiative rate *k*_*rad*_:

1 $$k_{rad} = \frac{{\phi_{fl} }}{{\tau_{fl} }}$$

The radiative rate is related to the dipole strength |*µ*|^2^ of the emitting state via Einstein’s coefficient of spontaneous emission:

2 $$k_{rad} = \frac{{16\pi^{3} }}{{3\upvarepsilon _{0} {\text{hc}}^{3} }}\frac{{n^{3} }}{{\upvarepsilon _{{\text{r}}} }}\nu^{3} \left| \mu \right|^{2}$$

where *n* is the refractive index of the surroundings of the studied molecules, *ν* is the frequency of the transition, and ε_0_, ε_r_, h, and c are the permittivity of vacuum, dielectric constant, Planck’s constant, and speed of light, respectively^28^. By combining Eqs. (1) and (2) one can determine the emitting dipole strength using the experimentally determined parameters. If the dipole strength of an aggregate is larger than that of the monomer, it means that fluorescence originates from a state shared by more than one molecule and the system exhibits superradiance.

The fluorescence quantum yield can be measured either in an absolute way using an integrating sphere, or in a relative way by comparing to a fluorescence standard^35^. Fluorescence of BChl *c* aggregates (both in chlorosomes and artificially prepared) was found to be too weak to be measured in an integrating sphere and therefore we used a relative method based on the measurement of absorbance and total fluorescence of a concentration series^35,36^. This series was compared to a concentration series of a standard dye with a known quantum yield, in this case HITCI in ethanol^37^. The fluorescence intensity was then plotted versus the 1-T absorption at the excitation wavelength and the slopes were used to calculate the fluorescence quantum yield^36^

3 $$\phi_{x} = \phi_{HITCI} \frac{{Grad_{x} }}{{Grad_{HITCI} }}\left( {\frac{{n_{TRIS} }}{{n_{EtOH} }}} \right)^{2}$$

where *ϕ*_*x*_ is the fluorescence quantum yield of the tested sample x and *n* is the refractive index.

**Figure 2:**
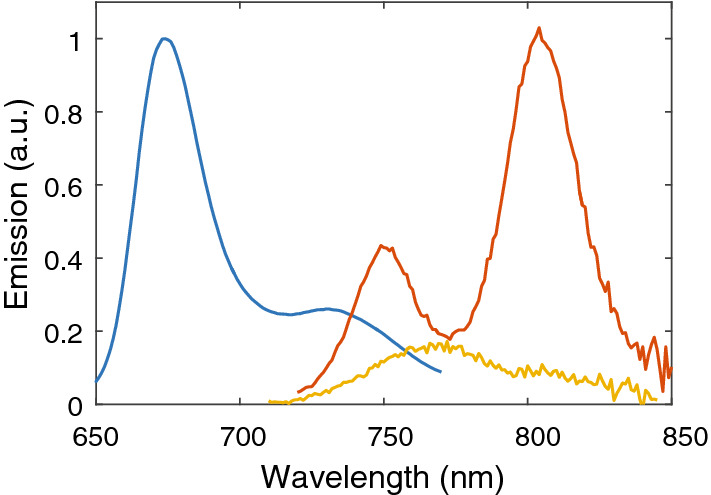
Steady-state fluorescence spectra of monomeric BChl *c* in ethanol (blue, excitation at 435 nm) and aggregated BChl *c* in chlorosomes from *Cba. tepidum* (yellow, excitation at 700 nm) and *Cfl. aurantiacus* (red, excitation at 710 nm) in a buffer at aerobic conditions. Both chlorosome spectra were multiplied by a factor of 40 for the sake of clarity.

Figure 2 compares the fluorescence spectra of monomeric BChl *c* in ethanol and BChl *c* aggregates in chlorosomes from both bacteria. The spectrum of the BChl *c* monomer consists of the 0–0 transition at ~ 675 nm and a redshifted vibronic satellite. The fluorescence shifts to ~ 750–770 nm upon aggregation of BChl *c*. Since chlorosomes contain also BChl *a*, which is the final acceptor of excitation energy located in the baseplate^6^, two fluorescence peaks are detected. BChl *a* emits around 805 nm. As mentioned in the Introduction, excitations are quenched at aerobic conditions in chlorosomes from *Cba. tepidum*, and to some extent also for *Cfl. aurantiacus.* The presented spectra were measured at aerobic conditions for reasons explained below, and therefore the overall fluorescence intensity and BChl *a*/BChl *c* intensity ratio are low in the case of *Cba*. *tepidum.* The emission spectra of the chlorosomes were fitted with two Gaussians to separate the two fluorescence peaks. The area under the Gaussian corresponding to BChl *c* aggregates was used as the fluorescence intensity.

**Figure 3:**
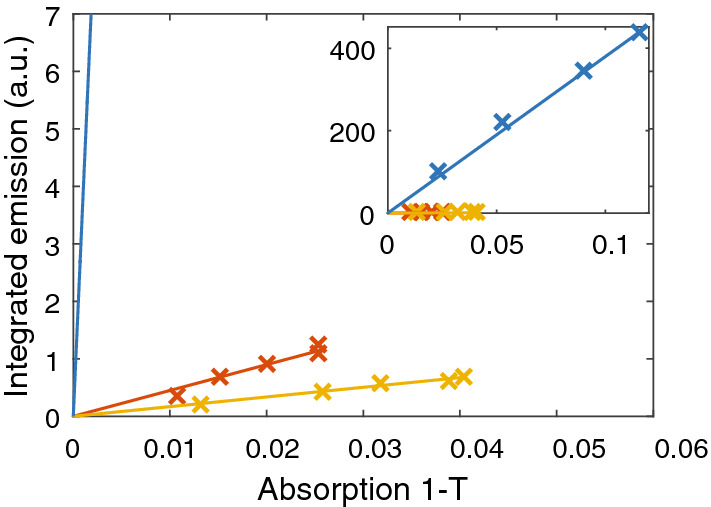
Fluorescence intensity integrated over all wavelengths as a function of the number of absorbed photons at the excitation wavelength for *Cfl. aurantiacus* (red line), *Cba. tepidum* (yellow line) and for BChl *c* monomers (blue line). The inset shows the data on an extended scale.

Figure 3 shows the gradients calculated according to Eq. (3). Using the data obtained for HITCI and its published quantum yield of 0.283^37^, the quantum yield of BChl *c* in ethanol was determined to be 0.213, which is in a good agreement with the published value of 0.21^38^. The same procedure was also applied for chlorosomes and artificial aggregates of BChl *c*. Table 1 summarizes the obtained quantum yields and demonstrates that aggregation leads to a substantial decrease of the fluorescence quantum yield by approximately two orders of magnitude.

**Table 1:** Quantum yields, lifetimes of the DAS components, emitting dipole strengths (calculated using $\varepsilon_{r} = n^{2}$ and $\lambda_{air} = \frac{c}{\nu }$ in Eq. (2) and combining Eqs. (1) and (2) into $\left| \mu \right|^{2} = \frac{{3{\upvarepsilon }_{{0}} {\text{h}}}}{{16{\uppi }^{3} }}\frac{{\lambda_{air}^{3} }}{n}\frac{{\phi_{fl} }}{{\tau_{fl} }}$) and their standard errors.

	BChl *c*: β-Car	Quant. yield	τ_1_ (ps)	τ_2_ (ps)	|µ|^2^ (D^2^)	Δ|µ|^2^ (D^2^)
BChl *c* monomers	1 : 0.0	0.213	5050	~ 200	31.4	2.9
"Slow-method" agg.	1 : 0.3	0.00203	32.4	7.2	78	8.2
"Fast-method" agg.	1 : 0.3	0.000638	22.2	9.6	33.2	3.8
*Cfl. aurantiacus*	– : –	0.00242	18.4	~ 80	148	29
*Cba. tepidum*	– : –	0.000946	21.1	~ 1.5	54.4	6.1

**Figure 4:**
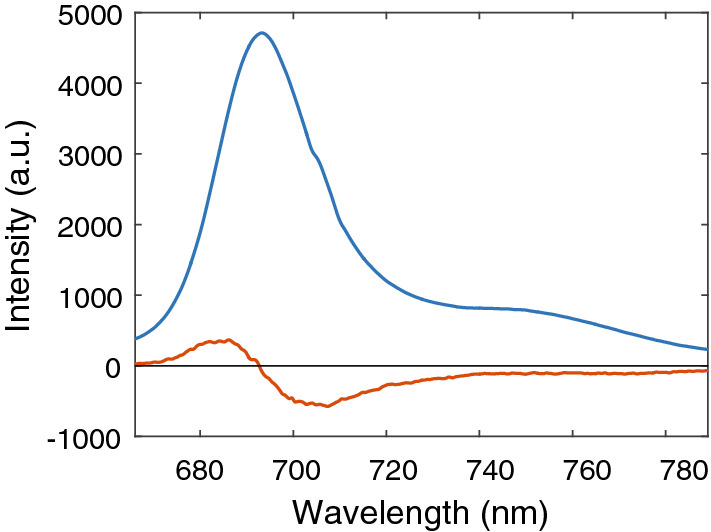
Decay-associated spectra of the two fluorescence decay components resolved for monomeric BChl* c*: 200 ps (red line) and 5050 ps (blue line).

**Figure 5:**
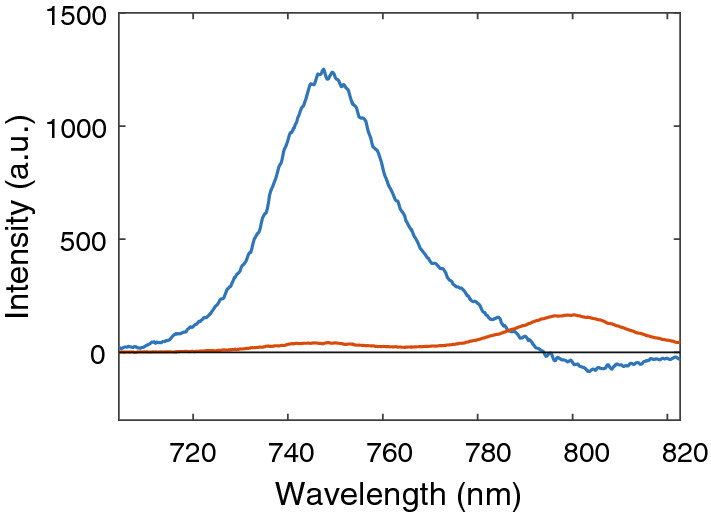
Decay-associated spectra of the two slowest fluorescence decay components resolved for the chlorosomes from *Cfl. aurantiacus* at aerobic conditions: 18.4 ps (blue line) and 234 ps (red line).

Fluorescence decay data measured with a streak camera (Supplementary Fig. S1) were analysed by global analysis and the results are presented here as decay associated spectra (DAS). The decay for BChl *c* monomers could be fitted with two lifetimes (Fig. 4). The fastest component has a lifetime of about 200 ps and exhibits a shape typical for solvent relaxation connected with population transfer from higher to lower energy states. The obtained fluorescence lifetime of 5.1 ns is typical for chlorophylls. The fastest DAS component for *Cfl. aurantiacus* chlorosomes has a lifetime of ~ 8 ps (not shown) and reflects EET from BChl *c* to BChl *a*. This lifetime is at the limit of our time resolution, and its value might not be accurate. Here we only consider the longer components, which largely describe the radiative decay. For *Cfl. aurantiacus*, the fluorescence decay of BChl *c* is described by a 18.4 ps lifetime (Fig. 5), i.e. two orders of magnitude shorter than the lifetime of monomeric BChl *c* .

The decay time of BChl *a* is much longer, 234 ps. Similar lifetimes of around 20 ps were also obtained for the decay of BChl *c* in chlorosomes from *Cba. tepidum* (Supplementary Fig. S2), while the decay of the BChl *a* fluorescence was much shorter than for *Cfl. aurantiacus,* 27 ps.

When the emitting dipole strength is calculated for BChl *c* in the chlorosomes from *Cfl. aurantiacus* using the lifetime of 18.4 ps and a quantum yield of 0.00242, a rather large value of ~ 150 D^2^ is obtained as compared to 31 D^2^ for BChl *c* monomers (Table 1). However, this value may be affected by back energy transfer from BChl *a* to BChl *c* and therefore not necessarily reflect a real strength of the emitting dipoles. Under physiological conditions, the excitation energy would be transferred from BChl *c* to BChl *a* in the baseplate and then to the next LHC in the energy transfer chain. However, in isolated chlorosomes a thermal equilibrium between populations of BChl *c* and BChl *a* is reached after the energy is transferred to the baseplate. The comparison of the DAS components for *Cfl. aurantiacus* (Fig. 5) with its steady-state fluorescence spectrum (Fig. 2) shows that the number of photons emitted with the 234 ps DAS is much higher than what would correspond to the ratio of the amplitudes of both DAS components. This 234 ps component also contributes substantially to the decay of BChl *c* in *Cfl. aurantiacus*. It is correct to use the lifetime of 18.4 ps to calculate the radiative rate of BChl *c*, because this lifetime represents the part of BChl *c* decay, which is not affected by the back energy transfer from (or equilibration with) BChl *a*. At the same time the fluorescence quantum yield of BChl *c* is affected (enlarged) by the back energy transfer (234 ps component), and this leads to an overestimated dipole strength for *Cfl. aurantiacus*. The apparent dipole strength in *Cfl. aurantiacus* chlorosomes increases 4.7 times as compared to that of monomeric BChl* c* but this value should thus be considered as an upper limit for the emission dipole strength.

Fortunately, the redox-dependent excitation quenching in *Cba. tepidum* allows for a realistic determination of the radiative rate for BChl *c* in chlorosomes. It should be noted that the presence of the quencher should not affect the radiative rate calculated by Eq. (1). The fluorescence lifetime is determined by the inverse sum of all the decay rate constants and the quantum yield is given by the same formula multiplied by the radiative rate constant. Thus redox-dependent excitation quenching, or any other decay channel, should affect both the lifetime and quantum yield to the same extent and their contributions in Eq. (1) should cancel out. Since the excitation is quenched already at the level of BChl *c*, a negligible EET to BChl *a* is expected and observed and therefore back energy transfer from BChl *a* to BChl *c* must be negligible as well. Direct excitation of BChl *a* is also negligible, since BChl *a* represents only ~ 1% of all BChls in the chlorosomes from *Cba. tepidum*^6^. Thus the enhancement of the dipole strength by a factor of 1.7 can be considered as a real value and it represents superradiance. The value may seem rather small, but it means that the emission originates from a delocalized state. Taking into account that this value was found to be equal to 2.8 and 3.8 (or even smaller, 2.1 and 2.9, after correction for a refractive index^39^) in very well ordered circular LH2 and LH1 complexes of purple bacteria^28^, the value of 1.7 found for self-assembling aggregates in chlorosomes represents a substantial enhancement.

To further check whether the determined extent of superradiance is realistic, we studied a simpler system of artificially prepared BChl *c* aggregates. These aggregates do not contain any BChl *a*, so no back energy transfer occurs. The aggregates also do not contain any quinones, which are responsible for the redox-dependent excitation quenching, although some extent of quenching is intrinsic to aggregates^10^. Carotenoids present in the aggregates do not affect the results significantly and their excitation energies are too high to allow for any back energy transfer. The fluorescence decay of all the aggregates could be fitted by two DAS components. The faster component was connected with internal relaxation and the slower, purely positive, reflects the radiative fluorescence decay of aggregates with a lifetime between 20 and 35 ps, i.e. similar as for BChl *c* aggregates in chlorosomes (Supplementary Fig. S2 and S4).

Fluorescence quantum yields were systematically larger in aggregates prepared by the "slow" method as compared to "fast" method aggregates. Since the lifetimes were similar, this leads to the occurrence of superradiance only for the ''slow" method aggregates. These aggregates exhibit a dipole strength even larger than for aggregates in chlorosomes of *Cba. tepidum* and two–threefold larger than for monomers and fast-method aggregates (Table 1 and Supplementary Table S1). It may be hypothesized that aggregates formed during the slow solvent exchange develop into more ordered and tightly packed assemblies, which leads to stronger excitonic interactions between pigments, compared to aggregates prepared by the rapid injection into a buffer. This is supported by a more pronounced redshift of the Q_y_ band for slow-method aggregates.

We can conclude that the strong coupling between the BChl *c* molecules in chlorosomes maintains excitation delocalization over at least two molecules even at the time of emission. This increases the dipole strength of the states from which EET (and emission) occurs and contributes to the high efficiency of energy transfer in chlorosomes. Even larger enhancement was observed for aggregates prepared by the "slow" method while no superradiance was observed for "fast" method aggregates. It should be noted that the dipole strength of the emitting states is still much smaller than that of the main absorbing states, which may be delocalized over larger parts of the aggregates. In conclusion, lowering the quantum yield of fluorescence by two orders of magnitude and, at the same time, increasing the dipole strength of the emitting states with respect to that of a monomer seems to be an important mechanism that increases the efficiency of EET between the coherent domains of BChl aggregates and towards BChl *a*. The mechanism might also be used to improve efficiency of EET in artificial devices for solar energy utilization based on artificial aggregates of BChl *c* as the source of excitation energy.

## Methods

*Cba. tepidum* and *Cfl. aurantiacus* J-10-fl cells were grown as described previously^24,33^ and chlorosomes were purified by ultracentrifugation using two sucrose gradients^40^. BChl *c* was isolated from *Cba. tepidum* as described in ref.^41^ and all four main homologues were pooled together and kept frozen. β-carotene was purchased from Sigma-Aldrich (Merck). Aggregates were prepared in 20 mM Tris–HCl buffer, pH 8.0 by two different methods. The first method is based on a rapid injection of a pigment mixture into aqueous buffer and vigorous shaking^33^, hence we call it the "fast" method. The mixture consisted of BChl *c* dissolved in ethanol and β-carotene in THF in a desired stoichiometric ratio. The second method is based on solvent exchange caused by slow addition of the buffer into the pigment mixture, this time both pigments dissolved in THF together with a block copolymer poly(butadiene-b-ethylene oxide) as described in ref.^34^ with modifications^24^. This method is referred to as the "slow" method. Aggregates were used 1–2 days after preparation, when they stopped developing as judged from absorption spectra. A more detailed description of aggregate preparation can be found in Supplementary material. In addition, solutions of monomeric BChl* c* and HITCI in ethanol were prepared as standards for the measurements of quantum yields and lifetimes. The used indices of refraction are described in Supplementary material.

Time-resolved fluorescence measurements were performed at room temperature with a synchroscan streak camera^42^ as described in ref.^43^. Spectral images of time- and wavelength-resolved fluorescence were obtained and corrected for background and for wavelength-dependent sensitivity of the detector. Processed streak images were then globally analysed using the Glotaran^44^ and TIMP package for R^45^ to determine the fluorescence lifetimes and decay-associated spectra (DAS) as described in ref.^3^. Samples were all excited at 400 nm with a Mira 900 laser (Coherent) operating at 800 nm and using a frequency doubler fs OPA-SHG (APE GmbH). The light intensity was modulated with neutral density filters to 50 μW at repetition rate of 253 kHz. Two measurement ranges were realised, one with a time resolution of 160 ps over 1024 pixels, another with a time resolution of 2.1 ns over 1024 pixels. The FWHM of the IRF was approximately 15 ps for the higher temporal resolution used and 30 ps for the lower resolution. Spectral resolution was approximately 5 nm.

To determine the fluorescence quantum yields of the samples, a concentration series was measured for absorbance using Agilent Cary4000 UV–Vis-NIR spectrophotometer with a full integrating sphere, and for total fluorescence intensity using Horiba Fluorolog 322 spectrofluorometer, respectively. This series has been compared to a concentration series of a standard dye with a known quantum yield, in this case HITCI in ethanol with a quantum yield of 0.283^37^. To cross-reference the precision of the measurements, monomeric BChl *c* with a known quantum yield of 0.21 in ethanol^38^ was measured as well. The fluorescence intensity was then plotted versus the number of absorbed photons at the excitation wavelength and the linear slopes were used for the calculations of the fluorescence quantum yield^36^. The absorbance of samples used for fluorescence measurements never exceeded a value of 0.15 in the peak of the Q_y_ band (1 cm path length). The fluorescence of chlorosomes was intentionally measured at aerobic conditions. The chlorosomes and artificial aggregates were excited at the blue end of the Q_y_ band to avoid excitation of β-carotene (700 nm for *Cba. tepidum*, 710 nm for *Cfl. aurantiacus*, 690 nm for aggregates prepared by the "fast" method and 705 nm for the "slow-method" aggregates). Monomeric BChl *c* was excited in the Soret band at 435 nm. This has no impact on the measured fluorescence, because relaxation from higher excited singlet levels to the first excited singlet level occurs with 100% efficiency and is several orders of magnitude faster than the fluorescence lifetime.

## Supplementary Information

Supplementary Information
