# Publisher Correction: Superradiance of bacteriochlorophyll *c* aggregates in chlorosomes of green photosynthetic bacteria

**DOI:** 10.1038/s41598-021-98623-3

**Published:** 2021-09-15

**Authors:** Tomáš Malina, Rob Koehorst, David Bína, Jakub Pšenčík, Herbert van Amerongen

**Affiliations:** 1 Department of Chemical Physics and Optics, Faculty of Mathematics and Physics, Charles University, Prague, Czech Republic; 2 Laboratory of Biophysics, Wageningen University, Wageningen, The Netherlands; 3 MicroSpectroscopy Research Facility, Wageningen University, Wageningen, The Netherlands; 4 Faculty of Science, University of South Bohemia, České Budějovice, Czech Republic; 5 Biology Centre, Czech Academy of Science, České Budějovice, Czech Republic

Correction to: *Scientific Reports* 10.1038/s41598-021-87664-3, published online 16 April 2021

The original version of this Article contained an error in Table 1, where the emitting dipole strength of “Fast-method” agg. was incorrectly given as “78”. The correct value is “33.2”.

The original Article has been corrected.

