# Supplementary material for: Superradiance of bacteriochlorophyll c aggregates in chlorosomes of green photosynthetic bacteria
Source: Sci Rep. 2021 Apr 16;11:8354. doi: 10.1038/s41598-021-87664-3 (PMC8052352; doi:10.1038/s41598-021-87664-3)
Supplement: Supplementary file 1 — Supplementary Information [file 41598_2021_87664_MOESM1_ESM.pdf]

Supplementary material

Superradiance of bacteriochlorophyll *c* aggregates in chlorosomes of green photosynthetic bacteria

Tomáš Malina[1], Rob Koehorst[2,3], David Bína[4,5], Jakub Pšenčík[1]* and Herbert van Amerongen[2,3]

[1]Department of Chemical Physics and Optics, Faculty of Mathematics and Physics, Charles University, Prague, Czech Republic
[2]Laboratory of Biophysics, Wageningen University, Wageningen, The Netherlands
[3]MicroSpectroscopy Research Facility, Wageningen University, Wageningen, The Netherlands
[4]Faculty of Science, University of South Bohemia, České Budějovice, Czech Republic
[5]Biology Centre, Czech Academy of Science, České Budějovice, Czech Republic
*corresponding author

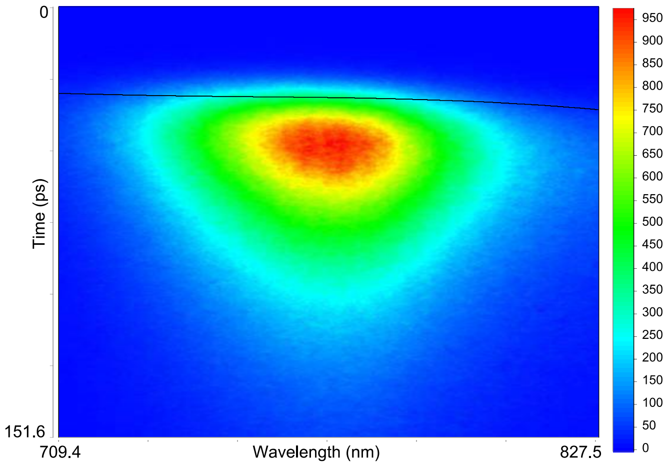

Figure S1
The spectrally and temporally resolved image from streak camera of "slow-method" aggregates with BChl *c* to β-carotene ratio of 1:0.3. Black curve corresponds to the time zero point at all wavelengths.

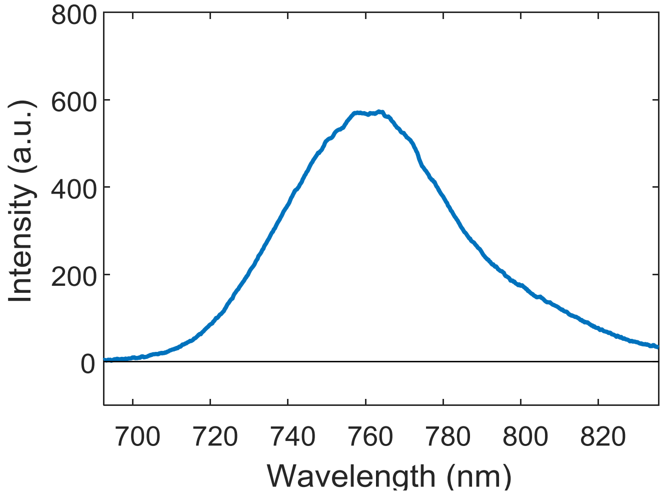

Figure S2
Decay-associated spectrum of the slowest fluorescence decay component resolved for the chlorosomes from *Cba. tepidum* at aerobic conditions: 20.2 ps. Due to the low intensity of the fluorescence signal, fitting of the data was difficult. Use of one component for fitting (as shown in the figure) led to relatively large residues, use of two components led to non-physical results.

Internal relaxation in aggregates
The faster component is different for the two types of aggregates. For "slow-method" aggregates, it is purely negative, smaller in amplitude (although not more than 30%), and slightly redshifted (approx. by 5 nm) as seen in Figure S3. It may be attributed to a relaxation from higher states, probably from the Soret band. It is present in all measured "slow-method" aggregates with a very similar relative amplitude, therefore it is not related to energy transfer from β-carotene to BChl *c*. For "fast-method" aggregates the faster component probably reflects exciton relaxation within the Q_y band.

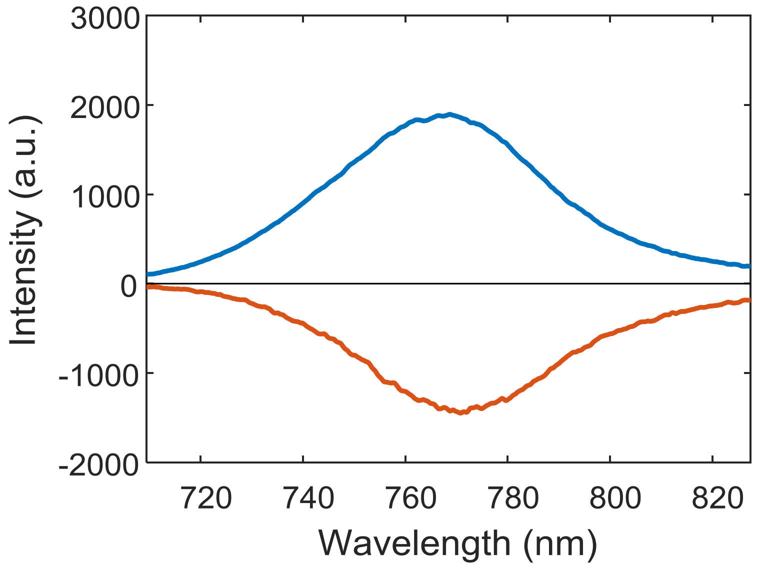

Figure S3
Decay associated spectra for two decay components of "slow-method" aggregates: 32.4 ps (blue line) and 7.2 ps (orange line). BChl *c* to β-carotene stoichiometric ratio was 1:0.3.

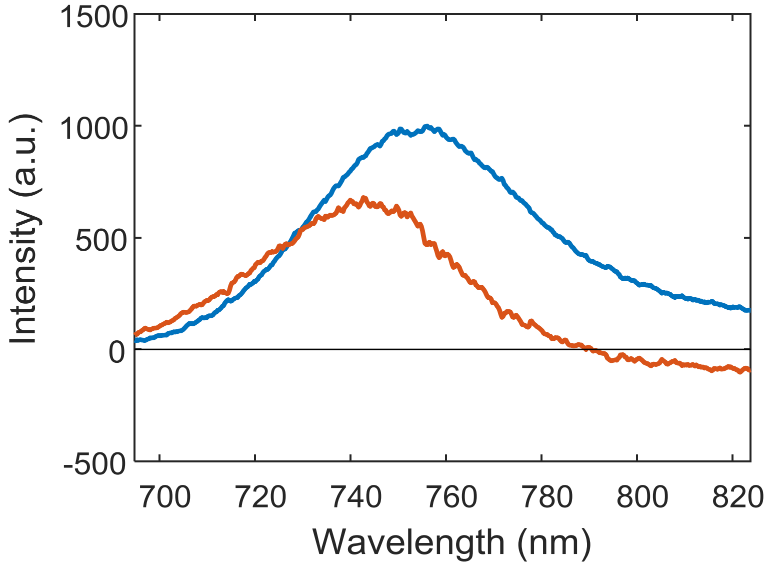

Figure S4
Decay associated spectra for two decay components of "fast-method" aggregates: 22.2 ps (blue line) and 9.6 ps (orange line). BChl *c* to β-carotene stoichiometric ratio was 1:0.3.

Effects of β-carotene on fluorescence lifetimes, quantum yields, and superradiance

Artificial aggregates were prepared by both the "slow" and "fast" method with stoichiometric ratios of BChl *c* to β-carotene between 1:0 to 1:1. Depending on the concentration of β-carotene different values for fluorescence quantum yield and lifetime were measured. The quantum yield of both types of aggregates tends to increase with the increasing amount of β-carotene incorporated into the structure (Fig. S5). The fluorescence lifetime increases with the addition of β-carotene as well (Fig. S6). Both these effects seem to be more prominent at lower concentrations of β-carotene (i.e. the increase of BChl *c* to β-carotene ratio from 1:0 to 1:0.5 yields a more significant change in both fluorescence lifetime and quantum yield than the increase from 1:0.5 to 1:1). Since the dipole strength is proportional to the ratio of the fluorescence quantum yield and the lifetime, the resulting change in the dipole strength (and therefore also superradiance) depends on which of these quantities changes more prominently. As a result, the dipole strength for "slow-method" aggregates tends to decrease with the increasing concentration of β-carotene while it increases for the "fast-method" aggregates (Fig. S7). The reason for the opposite effects can stem from a single cause – disorder. "Fast-method" aggregates are more disordered in general mostly due to the way of preparation which is very fast and leads to random arrangement of the aggregates. Adding more β-carotene to the mixture improves the long-range order of the pigments and leads to stronger interactions between molecules (as judged from an increasing redshift of the Q_y band, Fig. S9) resulting in a larger dipole strength. This is caused by the lipophilic properties of β-carotene, which incorporates between the layers of BChl c molecule and strengthen the interaction by hydrophobic effect.

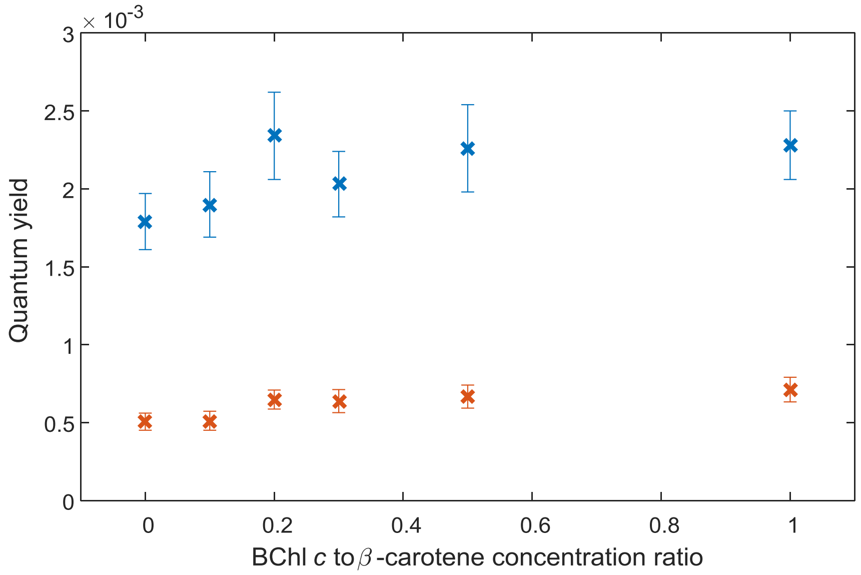

Figure S5

The quantum yield dependence on relative concentration of BChl *c* to β-carotene for both types of artificial aggregates ("slow-method" aggregates blue, "fast-method" aggregates orange).

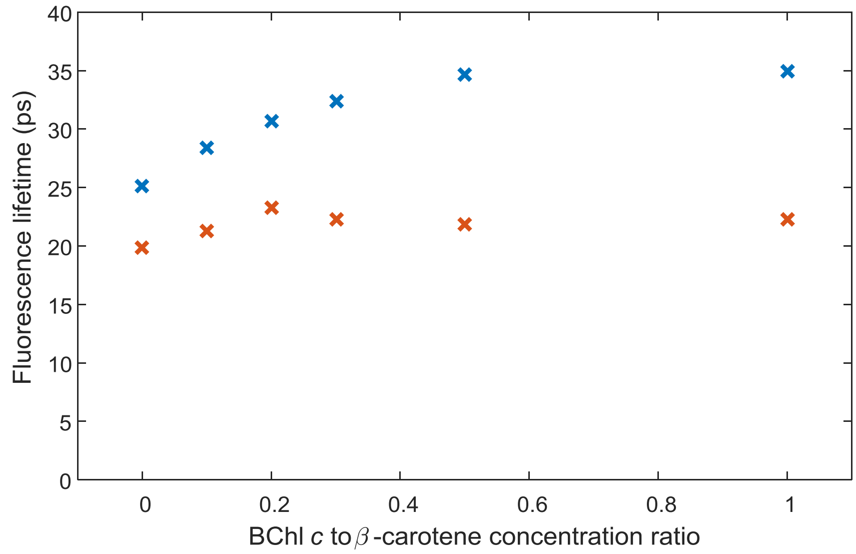

Figure S6
The fluorescence lifetime dependence on relative concentration of BChl *c* to β-carotene for both types of artificial aggregates ("slow-method" aggregates blue, "fast-method" aggregates orange). Error bars were too small to visualise.

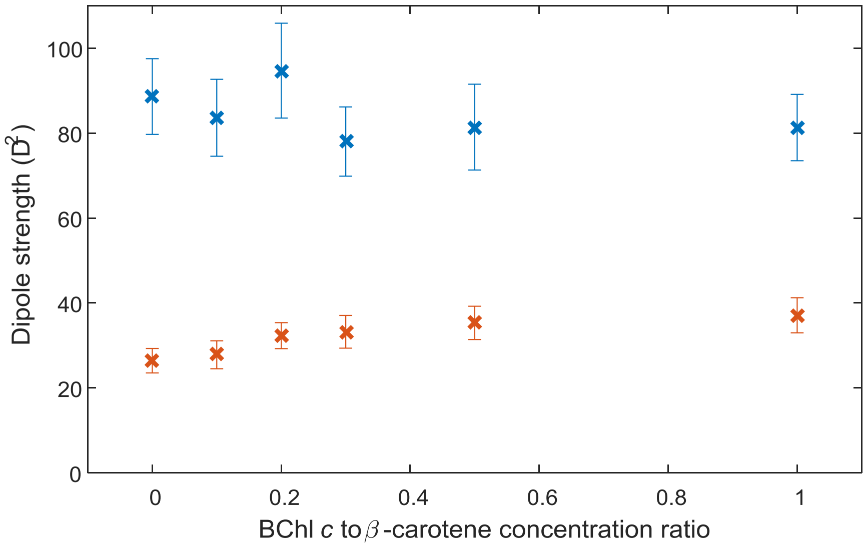

Figure S7
The dipole strength dependence on relative concentration of BChl *c* to β-carotene for both types of artificial aggregates ("slow-method" aggregates blue, "fast-method" aggregates orange).

On the other hand, incorporating more β-carotene into the "slow-method" aggregates may introduce disorder. During the "slow" method of preparation, BChl *c* molecules have a longer time to organise into more favourable orientation and produce aggregates with better long-range order. This is supported by the much higher value of the dipole strength. Addition of large amounts of β-carotene then may disrupt neatly stacked molecules of BChl *c*. This may induce disorder in their organization and decrease the dipole strength (as indicated by very slightly decreasing redshift with added β-carotene, Fig. S8).
Comparing the actual values of dipole strength leads to the conclusion that the extent of excitation delocalization at the time of emission is negligible in "fast-method" aggregates, while in aggregates prepared by the "slow" method the excitation appears to be delocalized over at least 3 molecules (Table S1).

Table S1
Quantum yields, lifetimes of the DAS components, emitting dipole strengths and their standard deviations (labelled as $\Delta|\mu|^2$) determined for artificial BChl *c* aggregates and BChl *c* monomers. τ_1 corresponds to the main fluorescence lifetime (positive peak in DAS).

	Bchl *c* : β-car	Quant. yield	τ_1 (ps)	τ_2 (ps)	$\|\mu\|^2$ (D^2)	$\Delta\|\mu\|^2$ (D^2)
"Slow-method" aggregates	1 : 0.0	0.00179	25.2	6.68	88.6	8.9
	1 : 0.1	0.00190	28.3	7.11	83.6	9.1
	1 : 0.2	0.00234	30.7	7.35	94.7	11.2
	1 : 0.3	0.00203	32.4	7.15	78	8.2
	1 : 0.5	0.00226	34.6	7.53	81.4	10.1
	1 : 1.0	0.00228	34.9	8.58	81.3	7.8
"Fast-method" aggregates	1 : 0.0	0.000506	19.9	11.2	26.4	2.9
	1 : 0.1	0.000512	21.2	10.5	27.8	3.3
	1 : 0.2	0.000648	23.2	12.1	32.3	3.1
	1 : 0.3	0.000638	22.2	9.59	33.2	3.8
	1 : 0.5	0.000667	21.9	9.02	35.3	3.9
	1 : 1.0	0.000712	22.2	8.87	37.1	4.1
BChl *c* monomers	1 : 0.0	0.213	5050	200	31.4	2.9

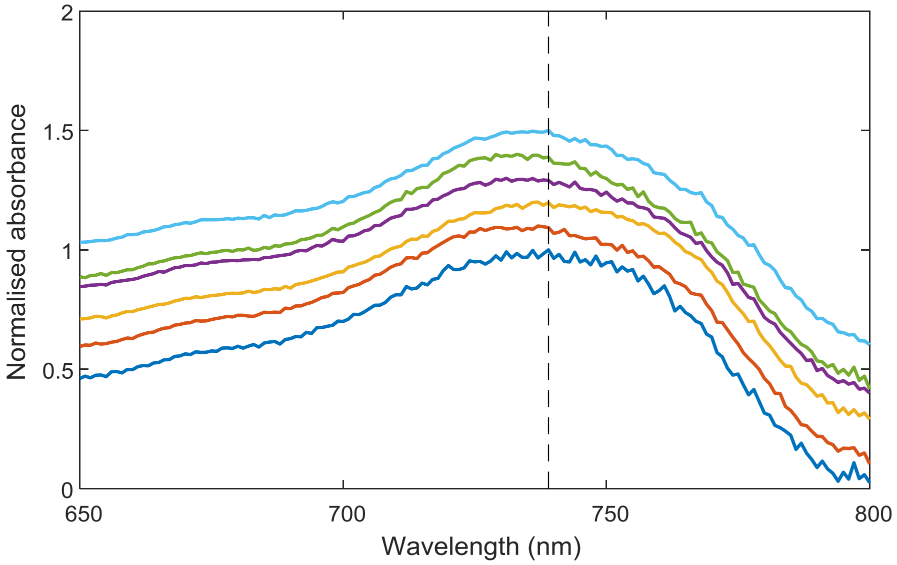

Figure S8
Absorption spectra of the Q_y bands of "slow-method" aggregates, each shifted vertically for the sake of clarity. Bottom curve represents BChl *c* to β-carotene stoichiometric ratio of 1:0, followed by curves for ratio of 1:0.1, 1:0.2, 1:0.3, 1:0.5, and 1:1.

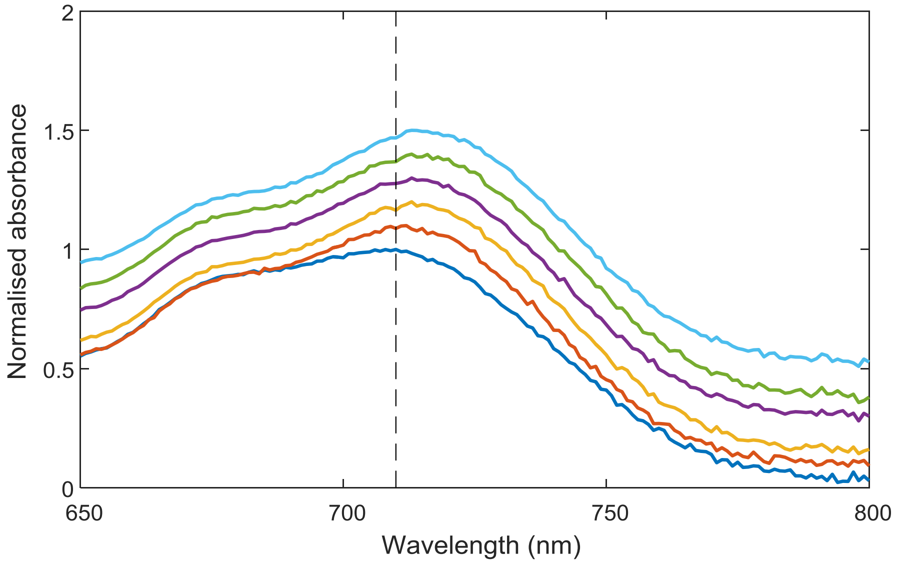

Figure S9
Absorption spectra of the Q_y bands of pure BChl *c* injected to a buffer by the "fast-method" (which forms probably dimers) and "fast-method" aggregates, each shifted vertically for the sake of clarity. Bottom curve represents BChl *c* to β-carotene stoichiometric ratio of 1:0 (BChl *c* dimers), followed by curves for ratio of 1:0.1, 1:0.2, 1:0.3, 1:0.5, and 1:1.

Preparation of artificial bacteriochlorophyll aggregates

All four homologs of BChl *c* isolated from *Cba. tepidum* were dissolved in ethanol. The concentration of the stock solution was between 2 and 5 mM. β-carotene was dissolved in tetrahydrofuran (THF) and the concentration of the stock solution was between 1 and 2 mM. The exact concentrations were determined spectroscopically using absorption coefficients 70 mM^{-1} and 140 mM^{-1} for BChl *c* (in the Q_y band maximum) and β-carotene, respectively.

Fast Method. The pigments were mixed in the desired stoichiometric ratio to yield samples with the final BChl *c* concentration of 15 μM in the buffer solution and a corresponding concentration of β-carotene. Samples were prepared in 2 ml of HCl-Tris buffer (20 mM, pH 8.0) by a rapid injection of the pigment mixture while vortexing. Samples were left in the dark for 24-48 hours at room temperature before the measurements to obtain steady-state absorption spectra. The resulting absorbance in the Q_y band maximum was around 0.3.

Slow Method. An aliquot of BChl *c* stock solution was used to obtain 15 μM BChl *c* in 2 ml. The solution was dried by nitrogen and re-dissolved in THF. Then, appropriate amounts of β-carotene were added to yield the desired concentration ratios. Poly(ethylene oxide)-block-poly(butadiene) (PEO-b-PBD, Polymer Source, Inc.) block co-polymer dissolved in THF was added in such an amount to obtain a molar ratio of 13.3:1 (BChl *c*:PEO-b-PBD). Additional THF was added so that the volume of the pigment and polymer mixture was approximately 80 μl to prevent drying of the mixture. The aggregates were prepared by slowly infusing the mixture with Tris-HCl buffer (20 mM, pH 8.0) at a rate of 2 ml per hour while stirring. After the preparation, the samples were left uncovered for 2-4 hours in the dark at room temperature in order to evaporate the remaining THF, and then they were stored in total for 24-48 hours before measurement. The resulting absorbance in the Q_y band maximum was around 0.5.

Index of refraction

Monomeric BChl *c* was measured in ethanol with an index of refraction of 1.36[1]. The index of refraction of the Tris-HCl buffer was assumed to be the same as that of water (1.33[1]) due to the low concentration of added salts and HCl. This value was used for pure BChl *c* injected into a buffer without any additions. For the aggregates, both in chlorosomes and artificially prepared, the buffer does not represent the immediate environment of the pigments. Therefore a value of 1.2 was used, which was determined elsewhere.[2]

Reference

1. Handbook of chemistry and physics. CRC Press, Boca Raton (2006).
2. Tang,K.H. *et al.* Temperature and Ionic Strength Effects on the Chlorosome Light-Harvesting Antenna Complex. *Langmuir* 27, 4816-4828 (2011).
